# Influence of combined therapy with conventional and herbal medicines on liver function in 138 inpatients with abnormal liver transaminase levels

**DOI:** 10.1186/s12906-016-1482-5

**Published:** 2016-12-01

**Authors:** Jun Hyuk Shin, Kyuseok Kim, Hae Jeong Nam

**Affiliations:** 1 Graduate school of Korean medicine, Kyung Hee University, #1 Hoegi-dong, Dongdaemun-gu, Seoul, 130-701 Republic of Korea; 2 Department of Ophthalmology, Otorhinolaryngology & Dermatology of Korean medicine, College of Korean Medicine, Kyung Hee University, #1 Hoegi-dong, Dongdaemun-gu, Seoul, 130-701 Republic of Korea

**Keywords:** Combined therapy of conventional and herbal medicines, Liver function, Liver transaminases, AST, ALT, GGT

## Abstract

**Background:**

To evaluate the influence of combined therapy of conventional and herbal medicines on liver function.

**Methods:**

This study was a retrospective chart review. A total of 138 patients with abnormal liver transaminase levels at the time of admission were included in this study. We evaluated the influence of combined therapy of conventional and herbal medicines on liver transaminase levels over a period of at least 2 weeks at Kyung Hee University Korean Medical Hospital. Analyses were performed using SPSS version 17.0 for Windows. Paired T-tests were used to examine the significance of differences in AST, ALT, and GGT levels at the time of admission and discharge.

**Results:**

We found that combined therapy reduced levels of aspartate aminotransferase (AST), alanine aminotransferase (ALT), and gamma glutamyl transferase (GGT) to a statistically significant level. Specifically, there were 48, 66, 104 subjects who exhibited abnormal AST, ALT and GGT levels at admission, which was reduced to 13, 37, and 64 subjects after combined therapy, respectively. Some subjects exhibited worsening levels of liver transaminases after combined therapy, so we used the χ^2^ test to analyze the influence of combined therapy with conventional and herbal medicines on liver function according to initial liver transaminase levels. According to this analysis, ALT and GGT levels may be more important than AST levels in estimating the influence of combined therapy on patients with abnormal liver transaminase levels.

**Conclusions:**

Based on this retrospective chart review, combined therapy of conventional and herbal medicines would be considered relatively safe. Thus, if patients have abnormal ALT or GGT levels, caution should be taken when suggesting combined therapy with conventional and herbal medicines.

## Background

The use of natural and herbal products for medical purposes has increased in many countries over the past few years. [1, 2] Some Asian countries, including Korea, even consider these natural and herbal products as medicines useful for preventing and treating various diseases. Indeed, many patients taking conventional medicine for diseases such as hypertension, diabetes, and hyperlipidemia also take herbal medicines.

As the interest in herbal medicines and supplements increases more and more, it has become necessary to verify the influence of herbal medicines on liver function. Specifically, concerns about hepatotoxicity caused by herbal medicines have been raised in several papers, but remains controversial. [3–6] Indeed, several other studies [7–10] have reported that herbal medicines are safe and, with respect to liver function, are more helpful than harmful. Given the growing prevalence of combined therapies of conventional and herbal medicines, a large-scale retrospective study was performed in Germany. [8] The authors of that study reported that 50.6% of 1,450 inpatients took herbal medicines at the same time as conventional Western medicine, of which 14 (0.97%) had drug-induced liver injury, although the prognosis of these patients was fine. Similarly, a study performed in Japan reported that only a small percentage of patients taking combined therapies of conventional and herbal medicines experience drug-induced liver injury due to herbal medicine. [9, 10]

A drawback of the studies described above is that they were performed using patients with normal liver transaminase levels, focusing only on hepatotoxicity. Thus, the influence of combined therapy of conventional and herbal medicines on liver function itself remains unclear.

To generate primary research with respect to evaluate the safety of combined therapy of conventional and herbal medicines on liver function, we selected hospitalized patients who had more than one abnormal value of aspartate aminotransferase (AST), alanine aminotransferase (ALT), and gamma glutamyl transferase (GGT) at the first examination. We then evaluated the influence of combined therapy (>2 weeks) on changes in AST, ALT and GGT levels.

## Methods

### Subjects

We analyzed patients who were hospitalized in Kyung Hee University Korean Medical Hospital. All patients were selected according to the following inclusion and exclusion criteria.

#### Inclusion criteria

① Patients who took herbal medicine combined with conventional medicine during hospitalization.

② Patients who had more than one abnormal value of AST, ALT, and GGT at first lab finding. AST > 40 U/L, ALT > 40 U/L, and/or GGT > 50 U/L.

#### Exclusion criteria

① Patients who were lost to follow-up for AST, ALT, and GGT levels before discharge.

### Data collection and analysis

The general characteristics of the study subjects, information about medications, and AST, ALT, and GGT levels at admission and discharge were collected from charts and analyzed retrospectively. Categorical data are presented as ‘number (N) (%)’ while continuous-type data are presented as the ‘mean ± standard deviation (SD)’. Analyses were performed using SPSS version 17.0 for Windows. Paired T-tests were used to examine the significance of differences in AST, ALT, and GGT levels at the time of admission and discharge. All differences were considered significant at *P* < 0.05.

### Ethical issues

This study was approved by the Institutional Review Board of Kyung Hee University Korean Medical Hospital. (IRB No. KOMCGIRB 2013-135)

## Results

### General characteristics of study subjects

The study consisted of a total of 138 subjects, 82 of them were male and 56 were female. The mean age was 60.12 ± 14.72 years old. The average interval of liver function tests was 41.87 ± 33.32 days. The most frequent disease for admission was cerebrovascular disease (ex. cerebral hemorrhage, cerebral infarction, subarachnoid hemorrhage, etc.), followed by the musculo-skeletal disorder, the psychiatric disorder (ex. depression, insomnia, etc.), the peripheral nerve palsy (ex, Bell’s palsy, paralytic strabismus, etc.), the vertigo and other diseases (idiopathic sudden hearing loss, stomach pain, post-herpetic neuralgia, and acute hepatitis). The average number of comorbidities was 2.224. The most frequent comorbidity is hypertension, followed by diabetes mellitus, cardiovascular disorders, and hyperlipidemia. Total 58 subjects had hypertension and thirty, twenty-two, and fifteen subjects had these comorbidities respectively. Thirty nine subjects had no comorbidity. The average number of conventional medicines being taken by each patient was 6.25. The most frequently used conventional medicine comprised anti-hypertension drugs: 126 of total 138 patients were being treated with anti-hypertension drugs (52 calcium channel blockers, 30 β-adrenergic blockers, 29 angiotensin II receptor antagonists, and 15 diuretics). The second and the third frequently used conventional medicine was anticoagulants & antithrombotics and agents for the digestive system respectively, followed by antiepileptics & skeletal muscle relaxants, agent for arteriosclerosis, antidepressants & tranquilizers, antidiabetics, and antiplatelet. It is uncertain for most subjects to identify the cause of liver biochemistry test abnormalities. Only twenty six subjects had non-alcoholic fatty liver disease. Six subjects had chronic hepatitis B and four subjects had alcoholic liver disease. One subject admitted to hospital for acute hepatitis.

Table 1 illustrated clinical characteristics, primary diagnosis for admission, comorbidities, conventional medicines that were taken and cause of liver biochemistry test abnormalities of all subjects.

**Table 1:** clinical characteristics, primary diagnosis for admission, comorbidities, conventional medicines that were taken and cause of liver biochemistry test abnormalities

Item	Number (%)
Number of patients	138 (100)
Gender	Male 82 (59.42), Female 56 (40.58)
Age (mean ± SD)	60.12 ± 14.72
Days of liver function test interval	41.87 ± 33.32
Primary diagnosis	
Cerebrovascular disease	82 (59.42)
Musculo-skeletal disorders	17 (12.31)
Psychiatric disorders	15 (10.86)
Peripheral nerve palsy	13 (9.42)
Vertigo	6 (4.34)
Other diseases	5 (3.65)
Comorbiditis	
Average number of comorbiditis	2.224
Hypertension	58 (42.02)
Diabetes mellitus	30 (21.73)
Cardiovascular disorders	22 (15.94)
Hyperlipidemia	15 (10.86)
Others	12 (8.69)
None	39 (28.26)
Conventional medicines	
Average number of medicines being taken	6.25
Anti-hypertension	126 (91.30)
Anticoagulants & Antithrombotics	122 (88.40)
Agent for digestive system	109 (78.98)
Antiepileptics & Skeletal muscle relaxants	67 (48.55)
Agent for arteriosclerosis	66 (47.82)
Antidepressants & Tranquilizers	66 (47.82)
Antidiabetics	44 (31.88)
Antiplatelet	44 (31.88)
Other CNS drugs	39 (28.26)
Other circulatory agents	28 (20.28)
Agents for liver diseases	22 (15.94)
NSIDS	21 (15.21)
Others	
Cause of liver biochemistry test abnormalities	
Acute Hepatitis	1 (0.72)
Chronic hepatitis B	6 (4.35)
Chronic hepatitis C	0 (0)
Alcoholic liver disease	4 (2.90)
Non-alcoholic fatty liver disease	26 (18.84)
Others	101 (73.19)

### Comparison of liver transaminase levels before and after combined therapy with conventional and herbal medicines

The mean levels of AST, ALT, and GGT were significantly decreased during hospitalization. Specifically, the average AST level at admission and discharge was 45.87 ± 49.68(U/L) and 27.64 ± 15.42 (U/L), respectively. Likewise, the average ALT at admission and discharge was 54.42 ± 69.87 (U/L) and 33.09 ± 24.29 (U/L), while that of GGT was 97.89 ± 102.57 (U/L) and 63.46 ± 52.34 (U/L), respectively (Table 2).

**Table 2:** Comparison of AST, ALT and GGT levels of all subjects and each subject group with abnormal liver transaminase levels before and after combined therapy with conventional and herbal medicines

Comparison	Variables(unit)	N	Admission (Mean ± SD)	Discharge (Mean ± SD)	*P*-Value
AST, ALT and GGT levels among all subjects	AST(U/L)	138	45.87 ± 49.68	27.64 ± 15.42	<0.001*
	ALT(U/L)	138	54.42 ± 69.87	33.09 ± 24.29	<0.001*
	GGT(U/L)	138	97.89 ± 102.57	63.46 ± 52.34	<0.001*
AST, ALT and GGT of each subject group with abnormal liver transaminase levels	AST(U/L)	48	81.29 ± 71.75	34.71 ± 20.77	<0.001*
	ALT(U/L)	66	87.85 ± 89.65	42.18 ± 25.27	<0.001*
	GGT(U/L)	104	119.33 ± 109.86	72.72 ± 55.38	<0.001*

*Analyzed by paired *T*-test, significant: *P* < 0.05

A total of 48 subjects had abnormal AST at admission while 13 subjects showed abnormal AST after combined therapy. Among the 13 subjects who presented with an abnormal AST, 9 subjects showed higher levels and 4 subjects showed lower AST levels compared to admission after combined therapy. A total of 69 subjects had abnormal ALT at admission and 37 subjects exhibited abnormal ALT after combined therapy. Among the 37 subjects who presented with an abnormal ALT, 18 had a higher ALT level while 19 subjects had a lower ALT level after combined therapy. Lastly, 109 subjects had an abnormal GGT at admission, 64 of which had an abnormal GGT after combined therapy. Among the 64 subjects who continued to have an abnormal GGT, 27 actually had a higher GGT level while 37 had a lower GGT level after combined therapy compared to the time of admission.

From a statistical standpoint, the average AST level at admission was 81.29 ± 71.75 (U/L), which decreased to 34.71 ± 20.77 (U/L) after combined therapy, with a significance probability less than 0.001. Likewise, the average of admission ALT was 87.85 ± 89.65 (U/L), which decreased to 42.18 ± 25.27 (U/L) after combined therapy, with a significance probability less than 0.001. Finally, the average GGT level at admission was 119.33 ± 109.86 (U/L), which decreased to 72.72 ± 55.38 (U/L) after combined therapy GGT, the significance of which was lower than 0.001 (Table 2).

Considering the period of combined therapy with conventional medicine and herbal medicine, the levels of AST, ALT, and GGT in subjects belonging to the 2–4 week treatment group were significantly decreased (*P* = 0.003, *P* = 0.003, and *P* = 0.004, respectively). Likewise, for the 4–8 weeks group, AST and GGT levels were decreased significantly (*P* = 0.025 and *P* = 0.011, respectively), while the ALT level was not significantly changed. (*P* = 0.070). Similar to the 4–8 week group, in subjects who received combined therapy for more than 8 weeks, the levels of AST and GGT were significantly decreased (*P* = 0.013 and *P* = 0.001, respectively), while that of ALT was not significantly changed (*P* = 0.110) (Table 3).

**Table 3:** Comparison of AST, ALT, and GGT of subjects according to the length of combined therapy with conventional and herbal medicines

Duration	N	Variables(Unit)	Admission (Mean ± SD)	Discharge (Mean ± SD)	P-Value
2-4 weeks	58	AST(U/L)	47.55 ± 56.49	28.72 ± 18.29	0.003*
		ALT(U/L)	64.86 ± 79.30	36.29 ± 26.64	0.003*
		GGT(U/L)	101.79 ± 117.43	68.22 ± 54.09	0.004*
4-8 weeks	52	AST(U/L)	39.62 ± 31.30	28.96 ± 14.19	0.025*
		ALT(U/L)	41.83 ± 24.62	34.00 ± 25.60	0.070
		GGT(U/L)	93.23 ± 104.11	65.38 ± 53.90	0.011*
More than 8 weeks	28	AST(U/L)	54.00 ± 61.53	22.96 ± 9.57	0.013*
		ALT(U/L)	56.18 ± 99.01	24.79 ± 12.76	0.110
		GGT(U/L)	98.46 ± 61.04	50.04 ± 44.64	0.001*

*Analyzed by paired T-test, significant: *P* < 0.05

### The influence on AST, ALT, and GGT change after combined therapy with conventional and herbal medicine

Although above results of liver transaminase levels showed safety of combined therapy, some subjects exhibited worsening levels of liver transaminases after combined therapy, so we next used the χ^2^ test to analyze the influence of combined therapy with conventional and herbal medicines on liver function according to initial liver transaminase levels. Considering the categorization of the liver transaminases, the prevalence of abnormal ALT and GGT after the combined therapy with conventional and herbal medicines in the abnormal group at baseline was 6.55 and 4.02 times higher than in normal range group at baseline, respectively. (*P* = 0.000 and *P* = 0.002, respectively) (Table 4).

**Table 4:** The influence on AST, ALT and GGT change following combined therapy with conventional and herbal medicines

	After the combined therapy with conventional and herbal medicines N (%)				
Before the combined therapy with conventional and herbal medicines N (%)			Normal range	Abnormal range	P-value
	AST	Normal range	85 (61.6%)	6 (4.3%)	0.132
		Abnormal range	40 (29.0%)	7 (5.1%)	
	ALT	Normal range	65 (47.1%)	8 (5.8%)	0.000*
		Abnormal range	36 (26.1%)	29 (21.1%)	
	GGT	Normal range	27 (19.6%)	8 (5.8%)	0.002*
		Abnormal range	47 (34.1%)	56 (40.6%)	

*Analyzed by *χ* ^2^ test, significant: *P* < 0.05

## Discussion

A growing number of people are taking conventional medicines for the treatment of diseases like hypertension, hyperlipidemia, and diabetes, which has coincided with an increase in the use of herbal medicines. [1, 2] Therefore for safe use of combined therapy with conventional and herbal medicines, it is necessary to study the influence of combine therapy on patients, especially with respect to the liver.

Several previous Korean studies have analyzed the influence of herbal medicine or combined therapy with conventional and herbal medicines on liver function, reporting that herbal medicines can be considered safe for liver function.[11–13] Two other studies [11, 12] were performed using admitted patients similar to the present study; however, they did not distinguish between patients with normal and abnormal liver transaminase levels. Indeed, most of the study subjects in the aforementioned studies had normal liver transaminases, and no effort made to separate patients who took herbal medicines from those who took combined therapy. A cross-sectional study by Park et al [13] evaluated herbal medicine intake and abnormal liver function. In that study abnormal liver function was not related with history of herbal medicine intake, but was related with male sex and overweight status. Specifically, among a total of 497 patients, 49 had not taken any medicine, 219 patients had taken only conventional medicine, 72 patients had taken only herbal medicine, and 157 patients had taken combined therapy. However, the authors of that study compared only two groups as follows: 219 patients who had taken only conventional medicine and 72 patients who had taken only herbal medicine.

Patients with underlying liver disease have a higher morbidity of liver injury caused by medicines compared with healthy individuals. [14] We assumed that if combined therapy with conventional and herbal medicine is harmful for liver function, patients with abnormal liver transaminase levels could be sensitive to combined therapy. Therefore, we focused on admitted patients at our hospital with abnormal liver transaminase levels to more clearly study the influence of combined therapy on liver function. Conventional medicines, especially those for hypertension, hyperlipidemia, and diabetes mellitus, are typically taken long-term. Likewise, herbal medicines are typically taken long-term rather than short-term because their effect is slow to manifest. Thus, we limited our analysis to patients who had taken combined therapy longer than 2 weeks.

This study didn’t attempt to confirm the efficacy of combined therapy but to evaluate the influence of combined therapy on liver function. Usually we clinicians couldn’t check every single medicine that has taken by patients, whether it is conventional or herbal. Therefore we roughly checked conventional and herbal medicines.

Herbal medicines are usually composed of several different types of herbs. Thus, we also documented the specific herbs being taken by patients. A total of 202 different kinds of herbs were used among the 138 enrolled patients. The most frequently used herbs were *Rhemannia glutinosa* (Gaertner) Liboschitz and *Glycyrrhiza uralensis* Fisch, which were used by all 138 patients. In addition, there were seven herbs used by greater than 100 patients: *Poria cocos* (Schw.) Wolf, *Panax ginseng* C. A. Mey. (= *Panax schinseng* Nees), *Atractylodes macrocepha*-*la* Koidz, *Angelica gigas* Nakai, *Scutellaria baicalensis* Georgi, *Paeonia albiflora Pallas* var. *trichocarpa* Bunge, and *Zingiber officinale* Rosc. Finally, 90 of the 202 different herbs were used by less than 10 patients, and only 26 herbs were used by more than half of patients.

There are several herbs which were considered harmful for liver function. In Korea, Yun et al [7] and Park et al [15] reported that there some herbs, for example *Ephedra sinica* Stapf, *Scutellaria baicalensis* Georgi, *Dictamnus dasycarpus* Turcz., *Aconitum carmichaeli* Debx *(= Aconitum chinense* Sieb. et Zucc*.), Euphorbia kansui* Liou ex Wang, *Croton tiglium* Linne, *Pinellia ternata* (Thunb.) Breit., *Rheum palmatum* L., *Sinomenium acutum* Rehder et Wils., *Polygonum multiflorum* Thunb., *Valeriana fauriei* Briquet could be harmful for liver function. F. Stickel & D. Shouval [3], C. Korth [4], and E.S. Bjornsson [16] also reported about the herbs which could be potentially harmful for liver function. Some of the herbs in these studies, for example *Agastache rugosa* (Fisch. et Meyer) O. Kuntze, *Tussilago farfara* L. [3], *Chelidonium majus* L., *Cimicifuga heracleifolia* Kom. [4], *Plantago asiatica* L. [16] were used but the other herbs were not used for the subjects in this study.

Table 5 illustrated the herbs which could be harmful for liver function were used in this study.

**Table 5:** The number of cases prescribed herbs previously reported as hepatotoxic agents

Herbs	Commonly used for	Number of cases
*Glycyrrhiza uralensis* Fisch.	Essential herbs for herbal medicine	138
*Scutellaria baicalensis* Georgi	Fever	104
*Pinellia ternata* (Thunb.) Breit.	Vertigo, Nausea	84
*Rheum palmatum* L.	Constipation	60
*Cimicifuga heracleifolia* Kom.	Menopausal syndrome	52
*Coptis deltoidea* C.Y. Cheng et Hsiao	Fever	48
*Ephedra sinica* Stapf.	Edema, Obesity	39
*Agastache rugosa* (Fisch. et Meyer) O. Kuntze	Common cold, Blood circulation	39
*Plantago asiatica* L.	Constipation	30
*Aconitum carmichaeli* Debx	Heart stimulant	19
*Aconitum ciliare* DC.	Blood circulation	7
*Tussilago farfara* L.	Cough, Common cold	7
*Sinomenium acutum* Rehder et Wils*.*	Edema	6
*Corydalis ternata* Nakai	For extravasated blood	6
*Polygonum multiflorum* Thunb.	Adaptogen	4
*Chelidonium majus* L.	Fever	1

The present study represents a primary research study on the safety of combined therapy with respect to liver function. Despite the positive finding that the average AST, ALT, and GGT levels of the 138 subjects were significantly reduced, there were a few subjects who had abnormal liver transaminase levels after combined therapy. Thus, in order to confirm the main factors by which liver transaminases predict the influence of combined therapy on liver function, which will be useful for clinicians who are considering suggesting combined therapy with conventional and herbal medicines, we analyzed subjects with abnormal liver transaminases using the χ^2^ test. According to this analysis, ALT and GGT levels may be more important than AST levels in estimating the influence of combined therapy on patients with abnormal liver transaminase levels. Thus, if patients have abnormal ALT or GGT levels, caution should be taken when suggesting combined therapy with conventional and herbal medicines.

This study had some weak points. First, the subjects in this study consisted of individuals who were admitted to the hospital, and thus were able to eat a healthy diet, did not consume alcoholic drinks, and were able to rest. Under such circumstances it is likely that the liver may be better able to repair itself. Therefore, future studies should be conducted in an outpatient setting. Secondly, unlike conventional medicines, herbal medicines consist of natural herbs, which can vary significantly with respect to quality. Thus, herbal medicines made with cheap herbs contaminated by agricultural chemicals or heavy metals may be harmful to liver compared with products of a higher quality. Thirdly, this study was a primary retrospective research study. In order to more fully evaluate the influence of combine therapy on liver function, it will be necessary to perform a randomized control study. Finally, this finding is a short term observation and that data regarding long term outcomes would be necessary to reach more definitive conclusions.

## Conclusions

The results of this retrospective study suggest that combined therapy with conventional and herbal medicines is relatively safe on liver function. However, some patients had increased liver transaminase levels, highlighting the importance of routine lab examination to continuously monitor patients with abnormal liver transaminase levels.

## Abbreviations

AST: Aspartate aminotransferase
ALT: Alanine aminotransferase
GGT: Gamma glutamyl transferase
